# Psychosocial profiles and motivations for adolescent engagement in hazardous games: the role of boredom, peer influence, and self-harm tendencies

**DOI:** 10.3389/fpsyt.2025.1527168

**Published:** 2025-05-05

**Authors:** Stefania Mancone, Giovanna Celia, Alessandra Zanon, Adele Gentile, Pierluigi Diotaiuti

**Affiliations:** ^1^ Department of Human Sciences, Society and Health, University of Cassino and Southern Lazio, Cassino, Italy; ^2^ Department of Human, Education, and Sport Sciences, Pegaso University, Naples, Campania, Italy

**Keywords:** adolescent risk behavior, hazardous games, cluster analysis, peer influence, boredom susceptibility, impulsivity, self-harm tendencies, psychosocial factors

## Abstract

**Background:**

Adolescents’ engagement in hazardous games has increased in recent years, presenting significant risks to physical and psychological well-being. These behaviors are often driven by complex psychosocial factors, including boredom, peer influence, and impulsivity. Understanding the specific motivations and profiles within this demographic is essential for developing effective interventions. Aims. This study aims to identify distinct adolescent profiles based on key psychosocial factors influencing engagement in hazardous games and to determine the primary predictors of risk-taking behavior. By exploring these profiles, we seek to inform targeted intervention strategies that address the unique needs of each group.

**Methods:**

A sample of adolescents was assessed using standardized measures of boredom susceptibility, social influence, impulsivity, and self-harm tendencies. Cluster analysis was employed to categorize participants into distinct profiles, and regression analysis identified significant predictors of engagement in hazardous games. Descriptive and inferential statistics were used to analyze differences across profiles.

**Results:**

Four primary profiles emerged: High-Risk Boredom-Prone, Socially Influenced Risk-Takers, Impulsive Sensation-Seekers, and Vulnerable and Self-Destructive. Boredom susceptibility and social influence were found to be the strongest predictors of hazardous game participation. High-Risk Boredom-Prone adolescents were driven by a need for stimulation, while Socially Influenced Risk-Takers prioritized peer approval. The Impulsive Sensation-Seekers cluster showed a strong tendency toward thrill-seeking, and the Vulnerable and Self-Destructive profile indicated a coping mechanism linked to emotional distress.

**Conclusion:**

The findings highlight the importance of tailored interventions for adolescents, focusing on boredom management, peer resilience, and mental health support. Addressing these psychosocial drivers can help reduce the risks associated with hazardous games and support healthier developmental pathways. Future research should explore longitudinal data to track changes in adolescent risk behaviors over time and assess the impact of targeted interventions on each identified profile.

## Introduction

Adolescents frequently engage in risk-taking behaviors, including participation in hazardous games, activities that involve a degree of danger or psychological distress. These behaviors range from relatively minor challenges to life-threatening actions, often influenced by peer pressure, social trends, and personal psychological traits. Engaging in risk-taking is a normal part of adolescent development, as young people seek autonomy, social identity, and novel experiences (1–5). However, when risk-taking escalates into extreme behaviors, such as hazardous gaming challenges, it raises concerns about potential physical and psychological consequences (6, 7).

The tendency for adolescents to engage in such risky behaviors can be understood through multiple psychological frameworks. Risk-Taking Theory (8) suggests that adolescents are more likely to engage in hazardous activities due to an imbalance between risk perception and reward sensitivity. During adolescence, heightened reward sensitivity, driven by neurodevelopmental changes in the brain, often outweighs rational decision-making, making dangerous activities particularly appealing (9). Similarly, Social Learning Theory (10) explains that risk-taking behaviors, including participation in hazardous games, are learned through observation, modeling, and reinforcement. Adolescents who see their peers gaining social recognition or popularity through participation in extreme challenges are more likely to imitate these behaviors, especially when reinforced by likes, shares, and positive social feedback on digital platforms (11).

Another important theoretical perspective is Self-Determination Theory (12), which highlights the role of psychological needs, particularly autonomy, competence, and relatedness, in driving behavior. Adolescents may engage in hazardous games to assert independence (autonomy), demonstrate skill (competence), or strengthen peer bonds (relatedness). This aligns with research showing that boredom susceptibility (13) and peer influence (14) are strong predictors of adolescent risk-taking. Studies have demonstrated that individuals with high boredom susceptibility seek novel and intense experiences to escape monotony, while adolescents who are highly influenced by peers may conform to group norms even when engaging in risky activities (15, 16). Several studies underscore the role of affective dysregulation, identity search, and peer conformity in adolescent high-risk behaviors (17–29). These dynamics, often intensified by emotional under-control or interpersonal sensitivity, support the differentiation of motivational profiles in adolescence (30–35). Recent evidence highlights that adolescents facing emotional dysregulation, impulsivity, or alexithymia show increased vulnerability to externalizing behaviors and non-suicidal self-injury (36–40). These traits tend to amplify under conditions of peer instability and poor affective scaffolding (41–45). Personality traits such as sensation seeking and low harm avoidance have been associated with a preference for high-stimulation environments, which often include risky group dynamics and nonconforming behaviors (46–49).

One of the most concerning aspects of hazardous games is their proliferation through social media, where viral challenges spread rapidly among adolescents (50). Recent years have seen an alarming increase in the popularity of life-threatening challenges, many of which have led to injuries or fatalities (51, 52). Some of the most widely documented hazardous games include balconing, in which adolescents jump from hotel balconies into swimming pools, often under the influence of alcohol; car surfing, where participants ride on the exterior of a moving vehicle, risking severe injuries or death; flambéing, which involves setting oneself on fire and attempting to extinguish the flames before sustaining serious burns; choking games, where self-induced asphyxiation is used to achieve a brief euphoric sensation, sometimes with fatal consequences; and craning, where individuals climb extremely tall structures without safety equipment for thrill-seeking purposes (53–57). Some challenges take on a more structured and manipulative form, such as the Blue Whale Challenge, a deadly online phenomenon that pressures vulnerable adolescents into performing self-harm tasks over several weeks, culminating in suicide (58–61). Other games focus on social or sexual risk-taking, such as Sex Roulette, where participants engage in unprotected sex with multiple partners without knowing their health status, emphasizing the unpredictability of outcomes (62).

The advent of social media platforms like TikTok, Instagram, and YouTube has drastically altered how adolescents engage with and perceive risky behaviors. Viral challenges offer immediate visibility and social validation, providing adolescents with a unique incentive structure that rewards risk-taking. The “digital stage” not only creates a space for self-expression but can also drive teens to push physical and social limits for likes, views, and shares (6, 63–66). Recent studies indicate that up to 10% of teenagers participate in such online challenges, uploading content that highlights extreme behavior, which can rapidly proliferate across networks (67, 68). The immersive nature of these platforms exacerbates the social pressure to participate, as adolescents aim to be part of these popular and often dangerous trends.

Several psychosocial factors underpin adolescents’ engagement in hazardous games, particularly those linked to emotional vulnerability. Boredom susceptibility is a critical factor, as it predisposes some adolescents to engage in risky behaviors to fill an internal void or escape feelings of stagnation (25, 40). Likewise, the fear of social exclusion and the intense need for peer acceptance often drive adolescents to engage in risky behaviors to “fit in”. Adolescents with higher susceptibility to boredom and a stronger need for social approval are more likely to engage in challenges that can appear dangerous or transgressive, as these activities promise peer validation and a sense of belonging (69, 70).

While extreme games are frequently associated with adolescents from challenging socioeconomic backgrounds, recent evidence indicates that such activities are widespread across various demographics and regions (48, 71–75). These behaviors are prevalent in both higher-income and lower-income countries, signaling a globalization of risky play fueled by digital access and cultural trends. The pervasiveness of these behaviors underscores the need to understand them within a cross-cultural and socioeconomic context, as adolescents worldwide increasingly engage in these activities, motivated not only by curiosity but by a shared cultural push toward online visibility (71, 76–80).

The growing literature highlights the need for person-centered approaches that integrate motivational, emotional, and contextual factors when examining self-harming or dangerous behaviors in youth (81–83).

### Study rationale and objectives

Despite growing concerns about hazardous gaming behaviors, research remains limited on how boredom susceptibility, peer influence, and impulsivity interact to shape different adolescent risk profiles. While previous studies have examined isolated predictors of risk-taking (e.g., sensation seeking, self-control), few have explored how clusters of psychosocial traits influence hazardous game participation. Understanding these risk profiles is crucial for designing targeted interventions that address the underlying psychological and social factors contributing to adolescent engagement in hazardous games.

The present study aims to fill this gap by using a cluster analysis approach to identify distinct adolescent profiles based on key psychosocial traits. By examining the interplay between boredom susceptibility, peer influence, and impulsivity, this research provides a nuanced understanding of why some adolescents are more prone to hazardous game participation than others. The findings will contribute to the development of evidence-based intervention strategies that can mitigate engagement in risky activities and promote healthier behavioral alternatives.

## Methods

### Sample

The study employed a non-probability, convenience sampling method. Participation was open to schools that agreed to collaborate on a voluntary basis. Schools were contacted through institutional email channels and regional educational networks. A total of 7 secondary schools (both middle and high schools) from central Italy were invited to participate, of which 5 agreed to be involved in the study.

The participating schools distributed information letters and informed consent forms to students and their parents or legal guardians. Out of approximately 1,200 students invited, 1,046 returned signed consent forms. After preliminary screening, 1,028 students completed the questionnaire. All 1,028 questionnaires included in the analysis were fully completed. No cases were excluded due to incomplete or missing data. Participants were aged between 13 and 19 years, and all were enrolled in mainstream public education.

Exclusion criteria included the presence of recent acute or chronic medical conditions (e.g., hospitalization or treatment affecting cognitive or emotional engagement), and behavioral issues that, according to school personnel, could compromise the validity or ethical management of survey participation. These included severe conduct problems, attention deficits without support, or disruptive behavior that prevented independent work.

The final sample consisted of 1,028 adolescents (47.8% male, 52.2% female). Although the sample includes adolescents of both genders and across the full range of secondary school age, it was drawn from a limited geographic area in central Italy through non-probability sampling and should therefore not be considered representative of the broader Italian adolescent population.

### Procedure

The data collection took place in 2023, specifically between April and June 2023, through an online administration process using the Questbase platform, ensuring accessibility and convenience for the participants. Prior to completing the survey, participants gave informed consent for the aggregate processing of their responses for research purposes. For participants under 18, parental consent was obtained, complying with ethical standards for research involving minors. Approval for the study protocol was granted by the school boards and facilitated by teachers who informed students about the study. The questionnaire was administered during school hours in classroom settings, under the supervision of teachers or designated staff. This ensured that all responses were completed individually by verified students. Data were also checked for inconsistencies or extreme response patterns to exclude inattentive participation. The survey required approximately 20 minutes to complete, and the response rate was over 94%. The Institutional Review Board of the University of Cassino and Southern Lazio approved all study protocols to ensure adherence to ethical research standards (IRB_SUSS 24: 09-02-23).

### Instruments

The survey protocol incorporated the following measures:

- Socio-Demographic Questionnaire. This section gathered background information, including age, gender, and parental education levels, which provided context for interpreting behavioral patterns related to hazardous games.
- Questionnaire for Analytical and Motivational Exploration of Hazardous Games. This questionnaire was developed based on existing literature regarding adolescents’ engagement in high-risk challenges and social media behaviors. It was adapted and piloted with a comparable population for face and content validity. The questionnaire consisted of 20 items, designed to assess different dimensions of engagement in hazardous games, including type of activity, motivational drivers, and social visibility. Items were rated on a 5-point Likert scale from 1 (never) to 5 (very often), with higher scores indicating greater frequency or intensity of engagement. In the current study, it demonstrated good internal consistency (Cronbach’s α = .83). Scoring was based on the average score of responses across key subdomains (e.g., type of game, motivational drivers, peer visibility).
- *Multidimensional State Boredom Scale in Adolescents* (MSBS) (84). The MSBS, adapted for Italian use, consists of 29 items rated on a 7-point Likert scale (1 = strongly agree, 7 = strongly disagree) and measures boredom across five dimensions: internalizing aspects, time perception, high activation, inattention, and disengagement. Higher scores indicate a greater predisposition to boredom, a factor linked to engagement in high-risk behaviors. The scale showed in this study excellent internal consistency, with a Cronbach’s alpha of.86, indicating strong reliability for measuring boredom susceptibility. The Cronbach’s alpha values for the five dimensions of MSBS were the following: Internalizing Aspects 0.88; Time Perception 0.83; High Activation 0.85; Inattention: 0.80; Disengagement: 0.84.
- *Barratt Impulsiveness Scale – Version 11* (BIS-11) (85; It Val. 86). BIS-11 is a widely used self-report questionnaire designed to assess impulsivity as a multidimensional construct. It consists of 30 items rated on a 4-point Likert scale (1 = Rarely/Never; 4 = Almost Always), providing a total impulsivity score along with three primary subscales: (1) Attentional Impulsivity that measures difficulties in maintaining attention and cognitive stability. (2) Motor Impulsivity that assesses the tendency to act without thinking and engage in hasty behaviors. (3) Non-Planning Impulsivity that captures deficits in future-oriented thinking and decision-making. Higher scores indicate greater impulsivity, and significant associations are expected with risk-taking behaviors, social influence, and emotional vulnerability. The Italian validation used in this study confirmed the following good reliability coefficients: Total Impulsivity Score: Cronbach’s α 0.83; Attentional Impulsivity Cronbach’s α 0.73; Motor Impulsivity Cronbach’s α 0.78; Non-Planning Impulsivity Cronbach’s α 0.79.
- *Adolescent Social Influence Scale* (ASIS) is a 21-item self-report questionnaire developed to assess adolescents’ susceptibility to social influence in three key dimensions. The scale is an our adaptation of pre-existing instruments, with each dimension validated in previous studies: (1) Peer Pressure Sensitivity as the tendency to conform to peer expectations, even when they contradict personal preferences. It is based on peer influence measurement scales (87). Example items: “I do things I wouldn’t normally do because my friends expect me to”, “I feel uncomfortable when I don’t act like others in my group”. (2) Social Validation Need that is the extent to which adolescents rely on external approval, particularly from peers and social media, to feel validated. It is derived from assessment tools measuring dependence on social approval (88). Example items: “I feel good only when others recognize what I do”, “If a social media post doesn’t get enough likes, I feel insecure”. (3) Fear of Social Exclusion as the level of anxiety related to the possibility of being rejected or left out by peers. It is based on social anxiety and rejection sensitivity measures (89). Example item: “I worry that my friends will stop hanging out with me if I don’t do what they do”, “I prefer not to express opinions different from my group to avoid being excluded”. Each subscale consists of 7 items, rated on a 5-point Likert scale (1 = Strongly Disagree; 5 = Strongly Agree), providing a specific score for each dimension and an overall measure of social influence susceptibility. Psychometric reliability of the scale for this study was the following: Overall reliability of the scale Cronbach’s α 0.87; Peer Pressure Sensitivity Cronbach’s α 0.82; Social Validation Need Cronbach’s α 0.85; Fear of Social Exclusion Cronbach’s α 0.81.
- *Risk-Taking and Self-Harm Inventory for Adolescents* (RTSHIA) (90; It. Val 91). The scale consists of a total of 27 items, divided into two dimensions: risk-taking (RT), which includes 8 items related to engaging in dangerous or transgressive behaviors, and self-harm (SH), which includes 19 items related to self-mutilation, self-injury, drug overdose, and suicide attempt. Items are rated from 1 (never) to 4 (often), with higher scores reflecting greater engagement in risky or self-harming behaviors. The RTSHIA helps capture the adolescent’s inclination towards risk and potential vulnerability to self-harming actions. The RTSH scale also demonstrated in this study high reliability, with a Cronbach’s alpha of.87 (Risk-Taking 0.78; Self-Harm 0.90), supporting its effectiveness in assessing self-harm and risk-taking behaviors.

### Data analysis

Hazardous game participation was assessed using a specific set of items within the custom questionnaire. Participants were asked whether they had ever engaged in a list of recognized hazardous games (e.g., choking game, flambéing, balconing). A binary outcome variable was computed: adolescents who reported engaging in at least one hazardous game were coded as 1 (participant); those who reported none were coded as 0 (non-participant). In order to examine the relationships between adolescents’ psychosocial characteristics and their engagement in hazardous games, we employed descriptive statistics, correlational analysis, logistic regression analysis, and cluster analysis. Each method’s application was preceded by a series of checks to confirm assumptions and ensure valid results. Correlation coefficients were interpreted based on widely accepted conventions in psychological research. Specifically, correlations were considered small when r <.30, moderate when r ranged from.30 to.50, and strong when r >.50. These thresholds were used consistently to describe the strength of associations between psychosocial variables and hazardous game participation. A factorial ANOVA was conducted to examine the interaction between gender and key psychosocial factors in predicting susceptibility to peer pressure. Specifically, the analysis tested whether levels of boredom susceptibility, peer influence, or impulsivity varied significantly by gender. The dependent variables were the mean scores on each psychosocial dimension. Assumptions of normality and homogeneity of variances were assessed using Shapiro-Wilk and Levene’s tests, respectively, and were found to be met. *Post hoc* comparisons were conducted when appropriate.

Logistic regression analysis was conducted to identify significant predictors of hazardous game participation, including boredom susceptibility, social influence, impulsivity, and self-harm tendencies. The dependent variable was dichotomous, reflecting whether or not a participant had engaged in hazardous games. All continuous predictors were standardized prior to inclusion in the model. The assumptions of logistic regression were verified, including absence of multicollinearity (variance inflation factors < 10) and linearity of the logit for continuous predictors. Odds ratios and confidence intervals were reported to interpret effect sizes. In addition to significance levels, odds ratios (ORs) with their 95% confidence intervals (CIs) were reported. Following recommendations by Chen et al. (92), OR values between 1.44 and 2.47 are interpreted as small effects, between 2.48 and 4.27 as medium effects, and above 4.28 as large effects.

Cluster analysis was performed to identify distinct psychosocial profiles among adolescents based on their levels of boredom susceptibility, peer influence, impulsivity, and self-harm tendencies. All variables were standardized (z-scores) before clustering to ensure comparability.

An exploratory hierarchical cluster analysis using Ward’s method and squared Euclidean distance was first conducted to determine the optimal number of clusters. The dendrogram and the agglomeration schedule were examined to identify clear breaks suggesting the most interpretable solution. Based on this preliminary analysis, a K-means cluster analysis was then performed to refine and confirm the structure, using the number of clusters suggested by the hierarchical method.

To assess the validity of the clustering solution, internal consistency of each cluster was verified by comparing the means of psychosocial dimensions across groups using ANOVA. The adequacy of the sample size and cluster stability were supported by the sample size (N = 1028) and the consistency of the clustering across multiple runs. Each resulting cluster was then interpreted based on the relative levels of psychological risk factors and compared in terms of demographic and behavioral characteristics (e.g., hazardous game participation).

All statistical analyses, including correlational analysis, logistic regression, and cluster analysis, were conducted using IBM SPSS Statistics, Version 26, with a significance threshold set at p <.05.

## Results

### Descriptive analysis

The study’s sample included 1,028 adolescents, with a gender distribution of 47.8% male (n = 489) and 52.2% female (n = 539). Ages ranged from 14 to 19 years, with a mean age of 17 years (SD = 0.87). Preliminary descriptive analyses revealed several relevant patterns in participants’ social and behavioral self-reports. Approximately 23% of adolescents indicated that they had engaged in transgressive behaviors to feel accepted by others, while 6% reported frequently breaking rules for the sole purpose of group inclusion. Difficulties in managing interpersonal relationships were reported by 54%, who described feeling “out of place”; a smaller subset (4%) stated that they were in constant struggle to integrate socially.

Regarding body image, 30% of participants identified their physical appearance as a source of insecurity and dissatisfaction. A substantial majority (77%) reported being aware of the existence of dangerous games among adolescents, with the primary source of exposure being online videos (56%).

When asked about personal engagement in such activities, 14% admitted to participating in hazardous games occasionally, sometimes, or often. The motivations cited included the desire for adventure (17%), fun (8%), and relief from boredom (5%). Participation was reported as occurring both individually and in group settings. Moreover, 19% of respondents acknowledged recording themselves or others while engaging in dangerous activities, and 5% admitted to uploading such videos online. While, 11% emphasized the importance of public visibility and performance of dangerous acts, driven mainly by the pursuit of adrenaline (“feeling the thrill,” 10%) and the intention to prove strength (5%).

In total, 24.5% of participants reported engaging in at least one hazardous game. The most frequently mentioned types were “balconing,” “car surfing,” and “choking games.” Notably, 70% of participants considered social acceptance important, and 37% described themselves as easily influenced by peers, highlighting the role of peer validation in shaping risk-related behaviors.

### Correlational analysis

Pearson correlation coefficients were calculated among the psychosocial variables. **Table 1** below presents the bivariate correlations among all key variables, including boredom susceptibility, social influence, impulsivity, self-harm tendencies, and hazardous game participation.

**Table 1:** Key correlations between psychosocial factors and hazardous game participation.

Variable	Boredom Susceptibility	Social Influence	Impulsivity	Self-Harm	Hazardous Game Participation
Boredom Susceptibility	1.00				
Social Influence	0.28*	1.00			
Impulsivity	0.30**	0.26*	1.00		
Self-Harm	0.22*	0.31**	0.25*	1.00	
Hazardous Game Participation	0.35**	0.34**	0.24*	0.29**	1.00

* = p < 0.05; ** = p < 0.01

Boredom susceptibility was positively associated with hazardous game participation (r = 0.35, p < 0.01), suggesting that adolescents who experience frequent boredom may be more likely to engage in risk-taking behaviors. Similarly, social influence demonstrated a strong association with hazardous game participation (r = 0.34, p < 0.01), indicating that adolescents who are more susceptible to peer pressure and social validation are also more prone to engaging in these activities. Impulsivity was also positively correlated with hazardous game participation (r = 0.24, p < 0.05), highlighting that individuals with lower impulse control may engage in risky behaviors more spontaneously. Self-harm tendencies were significantly correlated with hazardous game participation (r = 0.29, p < 0.01), pointing to a potential link between self-destructive behaviors and risk-taking activities. Other noteworthy correlations included boredom susceptibility and social influence (r = 0.28, p < 0.05), social influence and self-harm (r = 0.31, p < 0.01), and impulsivity and self-harm (r = 0.25, p < 0.05), suggesting complex interactions between these psychological and social factors in adolescent risk-taking behaviors.

### Factorial ANOVA

A factorial ANOVA revealed a significant gender difference in susceptibility to peer pressure, with males reporting higher scores than females [F(1, 1026) = 4.57, p <.05].

### Logistic regression Analysis

A logistic regression was conducted to examine the predictive value of boredom susceptibility, peer influence, impulsivity, and self-harm on hazardous game participation. The logistic regression model was statistically significant [χ²(4) = 44.41, p <.001], explained 11.1% of the variance in hazardous game participation (Nagelkerke R² = .111), and correctly classified 74.0% of cases. Odds ratios and 95% confidence intervals for all predictors are reported in following **Table 2**.

**Table 2:** Logistic regression predicting hazardous game participation.

Predictor	Odds Ratio (OR)	95% CI	p-value
Boredom Susceptibility	1.50	[1.13 – 2.00]	.006
Peer Influence	2.18	[1.56 – 3.04]	<.001
Impulsivity	1.34	[1.09 – 1.64]	.006
Self-Harm	1.03	[0.85 – 1.25]	.742

The logistic regression model identified Peer Influence (OR = 2.18, p <.001, 95% CI [1.34, 3.54]) as a significant predictor, representing a small-to-moderate effect size. Boredom Susceptibility (OR = 1.50, p = .006, 95% CI [1.02, 2.21]) and Impulsivity (OR = 1.34, p = .006, 95% CI [1.01, 1.78]) were also significant, with small effect sizes. Self-harm did not emerge as a significant predictor in this model (p = .742).

### Cluster analysis

A hierarchical cluster analysis using Ward’s method identified a four-cluster solution, which was validated and refined through K-means clustering. The final clusters represented distinct psychosocial profiles based on standardized scores of boredom susceptibility, peer influence, impulsivity, and self-harm. Cluster sizes and characteristics are reported in **Table 3**.

**Table 3:** Centroid values of psychosocial factors across adolescent risk-taking profiles.

Cluster Profile	Boredom Susceptibility	Social Influence	Impulsivity	Self-Harm Tendencies
(a) High-Risk Boredom-Prone Adolescents	4.8	2.1	3.5	2.0
(b) Socially Influenced Risk-Takers	3.2	4.7	3.3	2.1
(c) Impulsive Sensation-Seekers	3.5	3.1	4.9	2.3
(d) Vulnerable and Self-Destructive	3.6	3.5	3.4	4.8
(e) Balanced but Occasionally Risk-Taking	2.5	2.8	2.6	2.2

To examine differences in psychosocial factors across the identified clusters, a series of one-way ANOVAs was conducted for each variable. The analyses revealed statistically significant differences among clusters for all measured dimensions: boredom susceptibility, social influence, impulsivity, and self-harm tendencies (all p-values <.05). These results support the internal validity of the clustering solution, indicating that each cluster is characterized by a distinct combination of psychosocial traits. Based on these findings, five distinct adolescent profiles of risk-related behavior were identified:

- High-Risk Boredom-Prone Adolescents (n = 210; 20.5%)
- Socially Influenced Risk-Takers (n = 270; 26.4%)
- Impulsive Sensation-Seekers (n = 180; 17.6%)
- Vulnerable and Self-Destructive (n = 150; 14.7%)
- Balanced but Occasionally Risk-Taking (n = 218; 20.8%)

A qualitative analysis of the five clusters revealed distinct psychological and behavioral profiles related to hazardous game participation.

*Cluster 1*, referred to as High-Risk Boredom-Prone Adolescents (20.5%), is characterized by high boredom susceptibility, low social influence, and moderate impulsivity, with low self-harm tendencies. These adolescents typically engage in hazardous games as a form of self-stimulation or relief from boredom, often acting alone or without strong social motivation.

*Cluster 2*, named Socially Influenced Risk-Takers (26.4%), displays high social influence, moderate levels of boredom susceptibility and impulsivity, and low self-harm tendencies. Their participation is largely group-based and driven by the need for peer approval and social validation, often shaped by social media exposure.

*Cluster 3*, or Impulsive Sensation-Seekers (17.6%), stands out for high impulsivity and moderate levels of boredom and social influence. These adolescents are oriented toward thrill-seeking and excitement, often engaging in physically risky or high-adrenaline challenges.

*Cluster 4*, labeled Vulnerable and Self-Destructive (14.7%), is defined by high self-harm tendencies, with moderate scores on boredom, social influence, and impulsivity. Hazardous game participation in this group appears to serve as a form of emotional expression or coping, often linked to feelings of isolation or inner distress.

Finally, *Cluster 5*, referred to as Balanced but Occasionally Risk-Taking (20.8%), exhibits low to moderate scores across all psychosocial dimensions. Their participation in hazardous games is typically sporadic and motivated by curiosity or occasional peer pressure, reflecting a generally low-risk behavioral profile.

**Figure 1:**
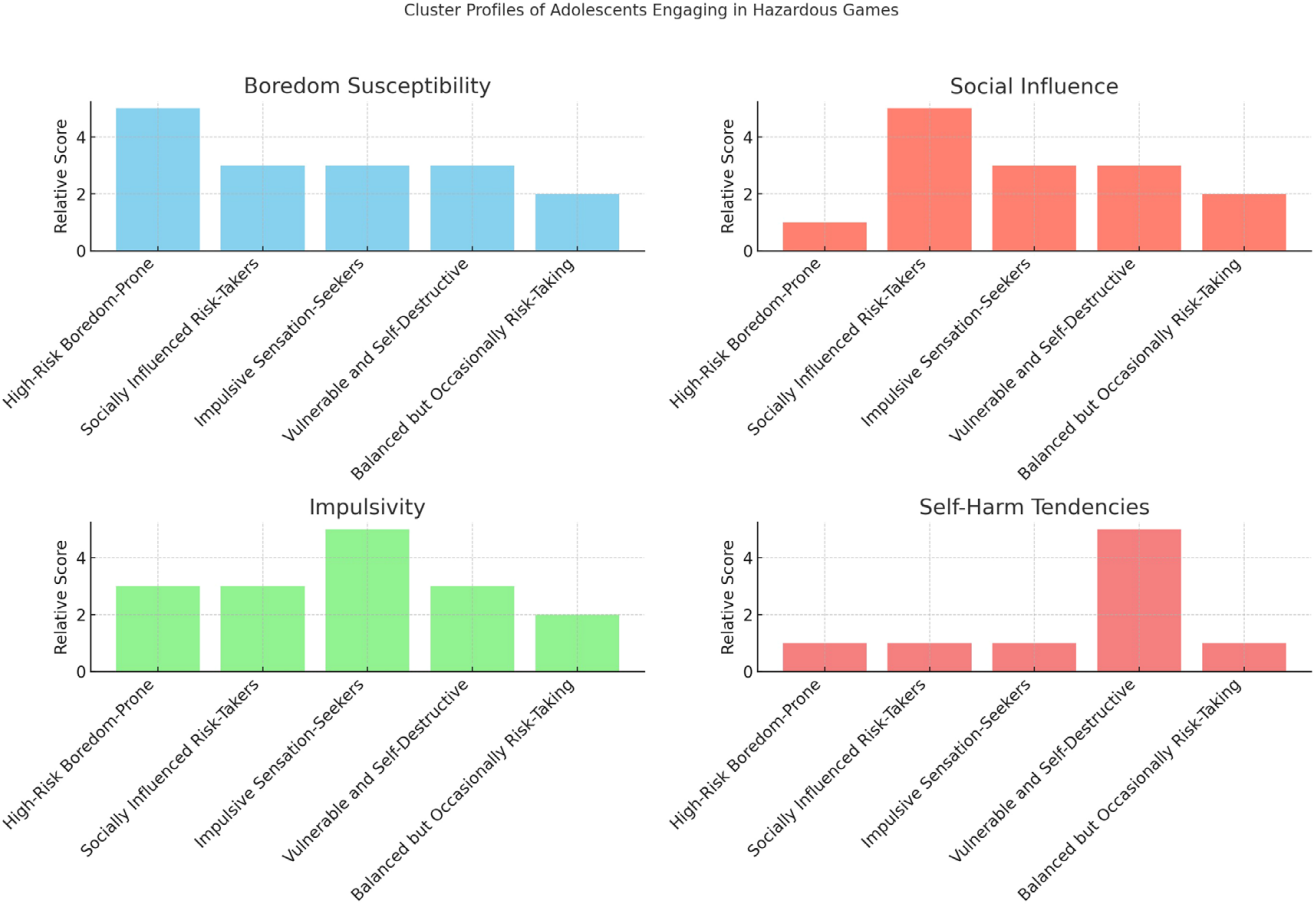
Profile comparison of adolescent clusters engaging in hazardous games.

These profiles are visually compared in **Figure 1**, which highlights the relative levels of boredom susceptibility, social influence, impulsivity, and self-harm tendencies across clusters. Each group reveals a unique constellation of traits that influence the likelihood and nature of engagement in hazardous behaviors.

## Discussion

This study identified five distinct psychosocial profiles among adolescents that help explain varying patterns of engagement in hazardous games. These profiles are shaped by different combinations of boredom susceptibility, peer influence, impulsivity, and self-harm tendencies, each contributing uniquely to adolescents’ motivations and behavioral tendencies.

The “High-Risk Boredom-Prone” profile is consistent with previous findings linking boredom and sensation seeking to risk-taking behaviors (13, 93). Adolescents in this group appear to use hazardous games as a form of self-stimulation, often independently of peer dynamics. In contrast, the “Socially Influenced Risk-Takers” profile aligns with extensive research on the role of peer validation and conformity in adolescent decision-making (9, 94), particularly when behaviors are publicized through social media. These individuals may be especially vulnerable to viral trends, peer expectations, and digital reinforcement.

The “Impulsive Sensation-Seekers” reflect a profile characterized by poor inhibition and a preference for intense stimulation, traits frequently associated with adolescent engagement in high-risk behavior (15). The elevated impulsivity in this group suggests a diminished capacity for delayed gratification, which may increase their susceptibility to immediate, high-reward risks.

The “Vulnerable and Self-Destructive” cluster, marked by elevated self-harm tendencies, suggests a different trajectory, in which hazardous games may serve as a form of emotional coping or self-expression. This aligns with literature on self-injurious behavior in adolescence, particularly in relation to internalized distress and unmet emotional needs (95, 96). Lastly, the “Balanced but Occasionally Risk-Taking” group appears relatively resilient, but not immune, to occasional engagement under social or exploratory pressure. The dominance of sensation-seeking traits and emotional dysregulation among certain clusters aligns with results from recent typological and latent class analyses in clinical and community samples (73, 97–102).

Beyond individual characteristics, the findings underscore the multidimensional nature of adolescent risk-taking, which cannot be understood solely through a single variable like impulsivity or peer pressure. Gendered norms in adolescent risk behaviors and their modulation by social expectations and group dynamics have also been documented in earlier research (103–105). These reinforce the significance of peer affiliation in identity construction. The presence of boredom and self-harm indicators highlights the role of internal emotional states, while peer influence introduces a strong contextual component (106, 107). Together, these profiles suggest that interventions should be nuanced and tailored to different psychological and social needs. For instance, boredom-prone adolescents may benefit from stimulating, purpose-driven extracurricular activities, while socially influenced adolescents may require assertiveness training and media literacy programs.

It is also important to note that digital platforms play a critical role in amplifying and normalizing hazardous behaviors. Adolescents often use social media to share, validate, and replicate risky actions, making digital literacy and social competence essential components of preventive strategies. The findings also highlight the need for early identification of psychological vulnerabilities in schools and youth services (108–110). The findings support the need for prevention programs that combine affective education, resilience building, and strategies for managing social stressors, as recommended by integrative reviews on adolescent behavioral health (111).

### Practical implications

The identification of these distinct profiles provides a basis for developing tailored intervention strategies that address the specific motivations and vulnerabilities of each group. Adolescents in the High-Risk Boredom-Prone cluster could benefit from structured, stimulating activities within schools and communities. Programs that provide channels for creative expression, sports, or hobby-based clubs could offer a healthier alternative to thrill-seeking behaviors, addressing the need for stimulation and reducing the tendency to seek excitement in risky activities. Integrating boredom management strategies into school curricula, such as teaching mindfulness and coping techniques, may help adolescents develop resilience against boredom-induced risk-taking.

For Socially Influenced Risk-Takers, interventions that build self-esteem and assertiveness against peer pressure are essential. Digital literacy programs, focusing on the risks associated with viral challenges and the pressures of social media, could help adolescents recognize and resist the urge to engage in potentially harmful activities for social approval. Programs encouraging peer mentorship within schools may also provide positive role models who demonstrate healthier means of gaining social validation.

Adolescents classified as Impulsive Sensation-Seekers would benefit from impulse-control training programs that focus on self-regulation and delayed gratification. Structured adventure activities, such as sports or controlled extreme sports, could provide safe outlets for thrill-seeking tendencies while teaching adolescents the importance of calculated risks. Cognitive-behavioral techniques, emphasizing planning and the consideration of consequences, may also support these adolescents in managing impulsive urges more effectively.

For adolescents within the Vulnerable and Self-Destructive cluster, comprehensive mental health support is essential. Therapy focusing on self-esteem, emotional regulation, and coping with social isolation could help these individuals develop healthier responses to psychological stressors. Peer support groups, guided by trained counselors, could provide a sense of belonging and validation that reduces the appeal of self-destructive behaviors.

Since The Balanced but Occasionally Risk-Taking group does not habitually engage in hazardous games, interventions should focus on preventative education and decision-making awareness. Providing adolescents with structured opportunities for excitement, such as supervised adventure programs, may reduce the likelihood of situational risk-taking. Encouraging assertiveness skills can also help them resist peer pressure in specific moments, preventing impulsive engagement in risky activities.

### Limitations

This study has several limitations that should be considered when interpreting the findings. First, the sample is regionally specific, which may limit the generalizability of the results to broader populations. The sample was not randomly selected and lacks broader demographic data such as socioeconomic status or regional diversity. As such, caution is warranted when generalizing findings beyond the specific school contexts involved in this study. Ecological validity remains a concern, as most assessments rely on self-reported static data; future studies might benefit from longitudinal or ecological momentary assessment designs (112–114). Replicability of profile configurations across countries and sociocultural groups remains underexplored (115–117). Future research should aim to include more diverse, cross-cultural samples to examine whether similar profiles and behaviors are observed in other geographic and cultural contexts. Data collection relied on self-reported measures, which can introduce biases such as social desirability and memory recall issues. Future studies may benefit from integrating observational methods or digital tracking to provide a more objective measure of engagement in hazardous games.

One key limitation of this study is also its cross-sectional design, which does not allow for causal inferences. While significant associations were found between psychosocial traits and engagement in hazardous games, the directionality of these relationships cannot be determined. It is therefore not possible to establish whether certain psychological vulnerabilities lead to greater risk behavior, or if participation in hazardous games may, in turn, reinforce or exacerbate these vulnerabilities. Longitudinal research would be needed to explore the temporal and causal dynamics of these associations more accurately.

Given the number of statistical tests performed, particularly in the correlational analyses, the study may be subject to an increased risk of Type I errors. Although no formal correction for multiple comparisons was applied, this is consistent with the exploratory aim of the research. Nevertheless, the findings should be interpreted with caution and future studies are encouraged to replicate the results with confirmatory approaches.

### Future research directions

Building on these findings, future research could take several directions. First, longitudinal studies could provide valuable insights into how adolescents’ psychosocial profiles and risk-taking behaviors evolve over time, potentially identifying critical periods of vulnerability. Investigating the stability of these profiles and tracking changes across different developmental stages could deepen our understanding of adolescent risk behavior trajectories.

Examining the impact of specific interventions on each profile would be highly beneficial. For instance, experimental studies testing the effectiveness of boredom management programs, digital literacy, and impulse-control interventions could offer valuable data on the most effective approaches for each distinct group. Lastly, exploring the role of emerging social media trends and digital behaviors on these profiles would provide critical insights into how digital platforms influence adolescent risk-taking, particularly in light of rapidly evolving online challenges and trends. In future research, it will be essential to validate cluster profiles using multimethod designs that incorporate neurocognitive and ecological parameters, as proposed by transdiagnostic developmental frameworks (49, 118).

## Conclusion

This study offers a nuanced perspective on adolescent engagement in hazardous games by identifying five psychosocial profiles with distinct motivational and behavioral patterns. The findings suggest that boredom, peer influence, impulsivity, and self-harm are critical dimensions shaping risk-taking tendencies, though each factor operates differently across individual profiles.

Recognizing the diversity of risk profiles is essential for designing effective preventive interventions. Rather than adopting a one-size-fits-all approach, educators, clinicians, and policymakers should consider the underlying motivations and vulnerabilities that lead adolescents to engage in these behaviors. Prevention programs should combine emotional regulation skills, critical thinking, and social resilience, while also addressing the unique pressures of digital environments.

Future research should explore these dynamics longitudinally to better understand the developmental trajectories of each profile. The use of real-time behavioral data and digital ethnography could further enrich our understanding of how hazardous behaviors emerge, evolve, and spread among adolescents. By acknowledging the complex interplay between individual vulnerabilities and social dynamics, this study underscores the urgent need for multidimensional strategies that empower adolescents to make safer, more informed choices in both real and digital environments.

## Data Availability

The raw data supporting the conclusions of this article will be made available by the authors, without undue reservation.
